# “Online survey of COVID-19 immunization and infection in patients with systemic juvenile idiopathic arthritis and adult-onset still’s disease.”

**DOI:** 10.1186/s12969-023-00911-x

**Published:** 2023-11-21

**Authors:** Mariana Correia Marques, Subrata Paul, Carol Lake, Ly-Lan Bergeron, Rashmi Sinha, Luciana Peixoto, Marinka Twilt, Michael J. Ombrello

**Affiliations:** 1 Translational Genetics and Genomics Section, National Institute of Arthritis and Musculoskeletal and Skin Diseases, Bethesda, MD USA; 2 Children’s National Hospital, Washington, DC USA; 3 Integrated Data Sciences Section, Research Technologies Branch, National Institute of Allergy and Infectious Diseases, NIH, Bethesda, MD 20892 USA; 4 Systemic JIA Foundation, Cincinnati, OH USA; 5 Section of Rheumatology, Department of Pediatrics, Alberta Children’s Hospital, Cumming School of Medicine, University of Calgary, Calgary, AB Canada

**Keywords:** COVID-19, Immunization, Vaccine, Systemic Juvenile idiopathic arthritis, Adult-onset Still’s Disease, sJIA, AOSD, SARS-CoV-2

## Abstract

**Background:**

Patients with systemic juvenile idiopathic arthritis (sJIA) and adult-onset Still’s disease (AOSD) have been under-represented in studies about safety of the COVID-19 immunization. We aimed to inquire about the safety and tolerability of COVID-19 immunization in this population.

**Methods:**

An anonymous online survey on closed Facebook groups for patients and parents with self-reported sJIA/AOSD was posted from June 27th until August 30th, 2022. Continuous variables were analyzed using t-tests or the Mann-Whitney U test if non-normally distributed. Fisher`s tests were used for categorical variables.

**Results:**

Of a total of 167 responses, 17 were excluded. Ninety-nine patients received the COVID-19 immunization, and 51 patients did not. Patients in both immunized and unimmunized groups had a similar history of disease complications such as macrophage activation syndrome (50% vs. 49%), lung disease (17% vs. 29%), arthritis (51% vs. 50%), and pericarditis/myocarditis (10% vs. 8%). Unimmunized patients were younger (median age 8 yo vs. 12 yo, p < 0.001) and had a higher incidence of a history of disease flare or severe side effects with other immunizations (24% vs. 4%, p < 0.001). Thirty-nine patients reported mostly mild immunization side effects. Severe side effects included 6 reports of disease flare and 2 reports of cardiac side effects (pericarditis and atrial fibrillation). Seven patients reported side effects lasting ≥ 8 days. Three patients developed AOSD following COVID-19 immunization, and 2 of them had the only hospital admissions for immunization side effects. Regarding COVID-19 infection, 46 patients were infected without full immunization, and 33 were infected after 2 doses of immunization. There was one hospitalization in the immunized group, compared to one ICU admission leading to death in the non-immunized group. There was a trend (p > 0.05) toward a higher risk of disease flare after COVID-19 infection among non-immunized patients (43%), compared to immunized patients (24%).

**Conclusions:**

The COVID-19 immunization was well tolerated by sJIA/AOSD patients even in this group of patients with severe disease. There was a low incidence of disease flare with immunization. Most immunization side effects were mild and lasted < 7 days. The only ICU admission and death from COVID-19 infection occurred in unimmunized subjects.

**Supplementary Information:**

The online version contains supplementary material available at 10.1186/s12969-023-00911-x.

## Key messages

- COVID-19 immunization was well tolerated for sJIA and AOSD, even with those with a severe phenotype.

- There was a small number of patients who had flares with the immunization.

## Background

It has been established that immunization is one of the most important tools for the control of the COVID-19 pandemic [1]. The safety and effectiveness of COVID-19 immunizations have been established for the general population, and some studies have been performed including patients with autoimmune conditions [2, 3]. However, patients with rare diseases such as systemic juvenile idiopathic arthritis (sJIA) and adult-onset Still’s disease (AOSD) were not well represented in these studies. The safety of COVID-19 immunization has been examined in AOSD patients with inactive disease, however, information about the safety of the immunization in children and in patients with more severe and active sJIA and AOSD is lacking [4]. In clinical practice and online patient forums, patients with sJIA and AOSD have expressed concerns about the COVID-19 immunization given the lack of information about these immunizations in these conditions. Patients also fear that the immunization would trigger a flare of their disease or cause significant side effects given their underlying immune system abnormalities. These fears have been compounded by the reports of subjects developing AOSD after receiving COVID-19 immunizations [5]. Therefore, we aimed to develop an anonymous online survey to gather information on the outcomes of COVID-19 immunization and infection in patients with sJIA/AOSD to help them make informed decisions regarding the risks and benefits of the immunization.

## Methods

Data were generated via an anonymous online survey using the SurveyMonkey platform with anonymous data collection. The survey was posted on closed Facebook groups from June 27th, 2022, until August 30th, 2022. The sJIA Arthritis Parents Network Facebook group has 1900 members and the Adult Onset Still’s disease group has 3300 members. Membership in these groups is curated by group coordinators and is limited to patients with sJIA or AOSD and their family members. The survey consisted of 27 close-ended questions in English and a final open-ended question that allowed respondents to make any further comments. Inclusion criteria included the age of respondents over 18 years and a reported diagnosis of sJIA or AOSD, regardless of immunization status and country of origin. If the patient was older than 18 years old, they could answer the survey for themselves otherwise it could be answered by their caregiver. The survey was terminated if respondents did not report a diagnosis of sJIA or AOSD or reported an age less than 18. Additionally, responses that failed to indicate whether the subject had received at least one dose of the COVID-19 immunization (immunization status) were excluded from further analysis. This study was conducted in compliance with the Helsinki Declaration and was exempted from review by the NIAMS IRB, (IRB# 000920) with a waiver of written informed consent. Consent to participate was requested at the beginning of the survey.

### Statistical analysis

Continuous variables were analyzed as medians and interquartile ranges (IQR). Categorical variables were analyzed as absolute numbers and percentages. Continuous variables were analyzed using t-tests or the Mann-Whitney U test when data were not normally distributed. Categorical variables were analyzed using Fisher’s exact test. Logistic regression was used to identify factors associated with flares with COVID-19 infection.

## Results

The survey received 167 responses. Seventeen responses were excluded from the analysis; 2 respondents were younger than 18 years old and 14 failed to answer the question about immunization status. One respondent had attested that they were older than 18 years old, but later marked that they were the patient, and selected a patient’s age younger than 18 years old. It was unclear which response was marked incorrectly, so we decided to exclude the patient from further analysis. Of the included patients, 131 had sJIA and 19 had AOSD. Among the 150 analyzed responses, 19 were from the affected subjects and 131 were from legal guardians responding on behalf of the patient.

### Patient characteristics

Patient demographics are displayed in Table 1, stratified by immunization status and by diagnosis. While the is a growing understanding that sJIA and AOSD are a part of the same disease continuum, the median age of the AOSD population was 40 years old (IQR 34,60), which was markedly different than the median age of the sJIA patients (9 years old, IQR 6,13). Therefore, to be able to differentiate particularities about the immunization for the sJIA patients, they were reported separately from the full cohort. There was only 19 AOSD patients, of whom only one was not immunized, which limited the ability to perform separate analysis to this group, so they were reported together with the full cohort.

Non-immunized sJIA participants were younger than the fully immunized (median age 7.5 vs. 11, p < 0.001) and had a younger age of onset of disease (median age of onset 2 vs. 5, p < 0.001). There was a higher proportion of female participants in the immunized sJIA group compared to the unimmunized (78% vs. 56%, p = 0.011). About 3/4 of the respondents were from the United States, and most others were from English-speaking countries. The rate of immunization among AOSD was 95% vs. 62% in sJIA. Patients in both unimmunized and immunized sJIA groups had a similar history of disease complications such as macrophage activation syndrome (50% vs. 56%), arthritis (52% vs. 53%), and pericarditis/myocarditis (8% vs. 10%). The incidence of lung disease trended higher in the unimmunized group, although it was not statistically significant (30% vs. 20%, p = 0.207). The unimmunized group reported more disease flares and severe side effects from other immunizations compared with the immunized group (24% vs. 1%, p < 0.001). Table 2 lists medications ever used to treat sJIA/AOSD. The most used medications to treat sJIA/AOSD reported by respondents were glucocorticoids and inhibitors of IL-1 or IL-6 (Table 2). Other less frequent medications reported by the patients, which are not listed in the chart, were IVIG (1 patient), colchicine (1 patient), sulfasalazine (2 patients), certolizumab (1 patient), ruxolitinib (1 patient), mycophenolate mofetil (1 patient), joint injections (2 patients).

On average, unimmunized sJIA subjects had treatment histories that included a higher number of different medications, excluding non-steroidal anti-inflammatory medications, than immunized patients (median 7 vs. 5, p = 0.049).

**Table 1:** Demographics

	Unvaccinated sJIA (n = 50)	Vaccinated sJIA (n = 81)			Unvaccinated sJIA + AOSD (n = 51)	Vaccinated sJIA + AOSD (n = 99)
Age (years)	7.5 (4, 9)***	11 (7, 14)***			8 (4, 9.5)***	12 (8,18)***
Age of onset (years)	2 (1, 4)***	5 (1, 9)***			2 (1, 4.5)***	7 (2, 12.5)***
Female Sex	28 (56%)*	63 (78%)*			29 (57%)*	79 (80%)*
Country						
USA	38 (76%)	65 (80%)			39 (76%)	73 (73%)
United Kingdom	1 (2%)	4 (5%)			1 (2%)	8 (8%)
Canada	1 (2%)	3 (4%)			1 (2%)	7 (7%)
Australia	3 (6%)	5 (6%)			3 (6%)	6 (6%)
Other	7 (14%)	4 (5%)			7 (14%)	5 (5%)
History of complications						
MAS	25 (50%)	45 (56%)			25 (49%)	49 (50%)
Lung Disease	15 (30%)	16 (20%)			15 (29%)	17 (17%)
Arthritis	26 (52%)	43 (53%)			26 (51%)	50 (50%)
Pericarditis/myocarditis	4 (8%)	8 (10%)			4 (8%)	10 (10%)
History of Disease flare or severe side effects with other immunizations	12 (24%)***	1 (1%)***			12 (24%)***	4 (4%)***
Total number of medications ever used beyond NSAIDs	7 (3, 8)*	5 (3, 7)*			7 (2.5, 8)*	4 (3, 6)*

*p ≤ 0.05,** p ≤ 0.01, ***p ≤ 0.001. Continuous values are represented as median (IQR). MAS: macrophage activation syndrome, sJIA: systemic juvenile idiopathic arthritis, AOSD: adult-onset Still’s disease, NSAIDs: non-steroidal anti-inflammatory drugs.

**Table 2:** Medications ever used to treat sJIA/AOSD

Medications ever used	Unvaccinated sJIA (n = 50)	Vaccinated sJIA (n = 81)		Unvaccinated sJIA + AOSD (n = 51)	Vaccinated sJIA + AOSD (n = 99)
NSAIDS	38 (76%)	71 (88%)		39 (76%)	83 (84%)
Steroids					
Oral	45 (90%)	70 (86%)		46 (90%)	88 (89%)
IV	35 (70%)	52 (64%)		35 (68%)	58 (59%)
IL-1 inhibitors					
Anakinra	41 (82%)	62 (76%)		41 (80%)	71 (72%)
Canakinumab	29 (58%)	50 (62%)		29 (56%)	51 (52%)
Rilonacept	2 (4%)	0 (0%)		2 (3%)	0 (0%)
Tocilizumab	33 (66%)	41 (51%)		33 (64%)	46 (46%)
Methotrexate	22 (44%)	36 (71%)		23 (45%)	45 (45%)
JAK inhibitors					
Baricitinib	0 (0%)	2 (2%)		0 (0%)	2 (2%)
Tofacitinib	20 (40%)	16 (20%)		20 (39%)	17 (17%)
Other JAKi	12 (24%)	7 (9%)		12 (23%)	8 (8%)
Cyclosporin	14 (28%)	25 (31%)		14 (27%)	26 (26%)
Tacrolimus	13 (26%)	10 (12%)		13 (25%)	11 (11%)
Abatacept	6 (12%)	7 (9%)		6 (11%)	9 (9%)
TNF inhibitors					
Adalimumab	5 (10%)	6 (7%)		5 (10%)	9 (9%)
Etanercept	5 (10%)	2 (2%)		5 (10%)	3 (3%)
Infliximab	3 (6%)	3 (4%)		3 (6%)	4 (4%)
Emapalumab	10 (20%)	2 (2%)		10 (20%)	2 (2%)
Cyclophosphamide	2 (4%)	3 (4%)		2 (4%)	3 (3%)
Azathioprine	0 (0%)	1 (1%)		0 (0%)	2 (2%)
Bone marrow transplant	1 (2%)	1 (1%)		1 (2%)	1 (1%)
IL-18 inhibitor	0 (0%)	1 (1%)		0 (0%)	1 (1%)
Hydroxychloroquine	1 (2%)	2 (2%)		1 (2%)	4 (4%)
Thalidomide/lenalidomide	0 (0%)	2 (2%)		0 (0%)	2 (2%)
Leflunomide	0 (0%)	4 (5%)		0 (0%)	4 (4%)

sJIA: systemic juvenile idiopathic arthritis, AOSD: adult-onset Still’s disease, NSAIDs: non-steroidal anti-inflammatory drugs, IL: interleukin, JAK: Janus kinase,

### Characteristics of immunized patients at the time of immunization

Table 3 lists the characteristics of immunized patients at the time of immunization. The majority of patients received the Pfizer/Biontech mRNA immunization (BNT162b2) (88% of sJIA and 78% of AOSD), followed by Moderna mRNA (12% of sJIA, 44% of AOSD), Oxford/Astra Zeneca (ChAdOx1) (1% of sJIA, 28% of AOSD%) immunizations, and Janssen (Johnson & Johnson, 2% of sJIA, 6% of AOSD). Approximately half of the patients reported inactive disease at the time of vaccination, while about a third had active disease when they were immunized. The most commonly used medications at the time of the immunization were oral steroids, IL-1, and IL-6 inhibitors. A complete list of medications used at the time of immunization is listed in Supplementary Table 1 in Additional File 1.

**Table 3:** Characteristics of immunized patients

	sJIA (n = 81)	AOSD (n = 18)
Which immunization*		
Pfizer/BioNTech	72 (88%)	14 (78%)
Moderna	10 (12%)	8 (44%)
Oxford/Astra Zeneca	1 (1%)	5 (28%)
Janssen (Johnson & Johnson)	2 (2%)	1 (6%)
Not sure	1 (1%)	0 (0%)
Did not answer	1 (1%)	0 (0%)
Disease activity level at the time of immunization		
Inactive Disease	42 (52%)	9 (50%)
Active Disease	31 (38%)	6 (33%)
Immunization before diagnosis	2 (2%)	3 (17%)
Other	5 (6%)	0 (0%)
Did not answer	1 (1%)	0 (0%)
How many doses of COVID-19 immunization		
1 dose	3 (4%)	0 (0%)
2 doses	38 (47%)	2 (11%)
3 doses	32 (40%)	6 (33%)
4 doses	7 (9%)	10 (56%)
Did not answer	1 (1%)	0 (0%)
Immunization side effects		
Yes	28 (34%)	11 (61%)
No	52 (64%)	7 (39%)
Did not answer	1 (1%)	0 (0%)

* Some patients received more than one type of immunization.

### Pausing medications for the immunization

The full list of medications paused for the immunization is listed in Supplementary Table 1 in Additional File 1. Twelve patients out of 99 who were immunized paused medication before immunization, including 6 who withheld methotrexate (60% of sJIA patients on methotrexate at the time of immunization), and one patient each who withheld oral glucocorticoids (6%), anakinra (20%), tofacitinib (11%), tacrolimus (20%), adalimumab (100%), and other JAK inhibitors (25%). One patient paused both leflunomide and tacrolimus for the immunization. Among the subjects who were on multiple treatments and who held some medications, several chose not to interrupt treatment with other medications before immunization, including tocilizumab (3 patients), NSAIDs (5 patients), hydroxychloroquine (2 patients), infliximab (1 patient), canakinumab (1 patient), and cyclosporine (1 patient).

### Side effects from immunization

Thirty-nine patients (39%) reported immunization side effects, which are listed in detail in Table 4. The most commonly reported side effects were local symptoms at the injection site, fever, chills, fatigue, myalgias, headache, nausea, lymphadenopathy, and rashes. Most side effects (79%) lasted 7 days or less, with the greater part lasting less than 2 days (41%). No medical care was required for the majority of patients. Thirteen patients reported severe and/or prolonged side effects, and they are described in detail in Supplementary Tables 2 and Supplementary Table 3 inside Additional File 1.

**Table 4:** Characteristics of immunization side effects

Characteristics of immunization side effects	sJIA (N = 28)	% of total immunized sJIA patients (N = 81)		AOSD (N = 11)	% of total immunized AOSD patients (N = 18)
Type of reaction					
Local symptoms at the injection site	20	25%		5	28%
Fever	13	16%		7	39%
Chills	7	9%		5	28%
Fatigue/ tiredness	21	26%		8	45%
Muscle pains	11	14%		6	33%
Headache	13	16%		6	33%
Nausea	3	4%		3	17%
Lymph node swelling	5	6%		3	17%
Rashes	3	4%		2	11%
Myocarditis	0	0%		0	0%
Disease flare	1	1%		2	11%
MIS-C/MIS-A	0	0		1	6%
Other (please specify)					
Atrial fibrillation	0	0%		1	6%
Pericarditis	1	1%		0	0%
Delayed flare	1	1%		0	0%
Flare of Crohn`s disease	1	1%		0	0%
Fever and URI symptoms	1	1%		0	0%
Food sensitivities	0	0%		1	6%
Coughing	1	1%		0	0%
Duration of reaction					
≤2 days	14	17%		2	11%
3–7 days	12	15%		3	17%
≥ 8 days	2	2%		5	28%
Did not answer	0	0%		1	6%
Highest level of care required for patients who had side effects					
No medical care required	16	20%		7	4%
Called physician	7	9%		2	11%
Visited a physician in clinic	3	4%		0	0%
Emergency department/urgent care	2	2%		0	
Hospital admission	0	0%		1	6%
Intensive care unit admission	0	0%		1	6%

### Pericarditis

One 12-year-old male patient required an emergency department visit without the need for hospital admission following Pfizer immunization. He reported having had pericarditis as a side effect of the immunization. He did not specify the timing of this complication relative to the immunization. This patient had a prior history of pericarditis/myocarditis as a complication of his disease. His disease had been inactive at the time of the immunization, and he was only being treated with NSAIDs at the time.

### Disease flares

A total of 6 patients (4 JIA and 2 AOSD) reported disease flares associated with the COVID-19 immunization. They are all listed in detail in Supplementary Table 2 on Additional File 1. Three patients marked “disease flare” as a side effect of the immunization. Three other patients reported disease flares as part of other comments of the survey. One patient (Patient 3), who did not report any side effects of the immunization, wrote in the comments that their child came out of remission 5 days after receiving the second dose of the COVID-19 immunization. The child is still having disease activity 7 months after the immunization. One patient reported a large flare 2 weeks after the second dose of the immunization. One patient reported a flare of Crohn`s disease after the immunization. Five out of the 6 patients who reported some type of flare with the immunization were on inactive disease at the time of immunization, and the majority of them did not hold medications for the immunization. Only the patient who had a flare of Crohn`s disease held methotrexate for the immunization but continued on infliximab. All the other 5 patients who flared did not hold their medications. Four of the 6 patients who flared with the immunization (patients 1, 2, 3, and 5) had a COVID-19 infection (3 after the second dose of the immunization, 1 after the third dose). All 4 of them also had a disease flare following the infection.

### New onset of AOSD following COVID-19 immunization

Three patients reported to have had COVID-19 immunization as the trigger for their disease onset, and two of them required hospitalization. These were the only hospitalizations for immunization side effects. They are described in more detail in Supplementary Table 3 in the Additional File 1. One 63-year-old male patient reported having developed a multisystem inflammatory disorder akin to AOSD following the administration of the Oxford/ Astra Zeneca immunization. He required an ICU admission and he also reported to have developed atrial fibrillation and multisystem inflammatory syndrome in adults (MIS-A). It is unclear if the diagnosis was MIS-A or the new onset of AOSD. This patient did not specify which dose of the immunization triggered the reaction. However, he did receive a total of 4 doses of the immunization, including Pfizer/ BioNTech, and he reported having used anakinra at the time of the boosters, and he reported that he did not have the disease before the first dose. He did not specify if subsequent doses of the immunization triggered flares. One 35-year-old female patient developed symptoms of AOSD a few days after her immunization with Oxford/ Astra Zeneca immunization. She required hospitalization for her symptoms. One 60-year-old female patient developed symptoms of AOSD 10 days after receiving a booster with Moderna immunization. The patients did not specify if they had flares or side effects with subsequent doses of the immunization.

### Reasons not to receive the COVID-19 immunization

Reasons cited by participants who decided not to receive a COVID-19 immunization are listed in Table 5. There was only one non-immunized AOSD patient, so all responses are reported together. The most common reasons were concerns about side effects from the immunization, both related to the diagnosis of sJIA/AOSD (29 patients, 58%), and unrelated to the diagnosis (23 patients, 46%). Sixteen (55%) participants who had concerns about immunization side effects related to sJIA/AOSD also had concerns about side effects not related to the patient`s condition. Fifteen out of 29 patients who had concerns specific to the underlying disease had a history of MAS, 9 had a history of sJIA-LD, and 3 had a history of pericarditis/myocarditis. Eleven patients reported concerns about the interaction of the immunization with their medications, including loss of effectiveness.

Six patients were told not to get the immunization by a medical provider. Four of these patients had a history of flares or severe side effects with other immunizations. Five of these 6 patients had a history of MAS, and 3 of the 5 also had a history of sJIA-LD. All of these 5 patients reported a history of multiple different immunomodulators, including IL-1 inhibitors, IL-6 inhibitors, tacrolimus, cyclosporin, and JAK inhibitors. Three of those patients had used emapalumab, and one of them had received a stem cell transplant. The patient who had no history of MAS was advised not to get the immunization due to long COVID-19 following infection.

Thirteen patients reported that the immunization was not available to the age group, and 1 patient reported an allergy to an immunization component. Eight patients reported other reasons for not receiving the immunization, which included one patient who reported a history of many severe medication allergies. One patient reported that they were told that they would need to stop the JAK inhibitors for one week before and one week after the immunization, and they were concerned with disease flare, so opted not to get the immunization. One patient believed that the benefit of the immunization did not outweigh the risk, citing the low risk of severe COVID-19 infection in pediatric patients. Two patients reported that the immunization had just become available to the age group and had not had a chance to get it yet.

**Table 5:** Reasons for not immunizing

Reasons not to get immunized (n = 50)	n(%)
Concern about potential side effects of the immunization that are not related to sJIA/AOSD	23 (46%)
Concern about potential side effects of the immunization related to the patient’s sJIA/AOSD	29 (58%)
Concern about interaction of the immunization with the patient’s medication (including loss of effectiveness)	11 (22%)
Immunization is not approved/not locally available for the patient’s age range	13 (26%)
Allergic to products in the immunization	1 (2%)
Told not to get COVID-19 immunization by a medical provider	6 (12%)
Other	8 (16%)

### Patients with a history of MAS or lung Disease

Of 75 patients who reported a history of MAS, 50 received at least one dose of the COVID-19 immunization. Sixteen patients had active disease at the time of the immunization, 29 patients had inactive disease, 4 patients were in medicated remission, and one patient had abnormal labs. The survey did not specify if the patient had MAS at the time of the immunization. Nineteen patients reported side effects from the immunization, which were mostly mild and lasted 7 days or less. Three patients reported some type of flare associated with the immunization, which are described in more detail in Supplementary Table 2 (patients 3, 4, and 5), and two patients had side effects lasting 8 days or more (patients 7 and 10). Of 33 patients with a history of sJIA-LD, 27 of them also had MAS. Characteristics of patients with a history of sJIA-LD are described in more detail in Supplementary Tables 4, stratified by immunization status. Of note, two children with sJIA- LD patients reported a history of a bone marrow transplant. There was only one AOSD patient who reported to have lung disease. This adult patient added as a free text that they had been diagnosed with a rare primary immune dysregulation disease, and also had immunologic changes as part of the presentation.

### COVID-19 Infection

Amongst the patients who answered the question about a COVID-19 infection, more patients tested positive for COVID-19 or had a diagnosis made by a medical provider in the unimmunized group compared with the fully immunized group (33/50, 66% vs. 46/95. 48%; p = 0.032). Three patients only received one dose of the immunization, and none of them had a COVID-19 infection. Some immunized patients contracted COVID-19 before receiving the immunization. Therefore, characteristics of the COVID-19 infection are listed in Table 6 stratifying patients according to the timing of the infection relative to the immunization and by diagnosis. Most patients had asymptomatic or mild/moderate symptoms. There was one ICU admission, which later resulted in death in a non-immunized patient, and there was one hospitalization to the general ward of an immunized patient. Patients with severe outcomes of COVID-19 infections are described in more detail in Supplementary Table 5. In the free comments section, five patients (patients A, B, D, F, and I) reported prolonged symptoms after a COVID-19 infection or reported having been diagnosed with long COVID in the comments. This was not specifically asked in the survey, so it is possible that more patients had this complication. Four of these patients contracted COVID-19 after having received a full immunization course.

### MIS-C/ MIS-A

Eight sJIA patients reported developing MIS-C/MIS-A following a COVID-19 infection (6 of them had contracted COVID-19 without any prior dose of the immunization, and 2 patients contracted the infection after immunization with the Pfizer/BioNTech immunization, 1 patient after the second dose and 1 after the third.) The survey did not specify the dates of the infection, so it is not possible to determine the strain that caused it. These patients had a median age of 7.5 yo (IQR 2.5yo). One outlier patient was a 22 yo male. Seven of these patients had a history of MAS, and 4 of them had a history of lung disease. Seven of these 8 patients also reported having flared of the disease with the COVID-19 infection. Two patients (a 6 and a 4-year-old patient) reported that COVID-19 triggered the development of sJIA. One of these patients specified that the disease was diagnosed 6 weeks after the COVID-19 infection.

**Table 6:** Outcomes of COVID-19 infection (N = 79)

	Asymptomatic	Mild/Moderate	Hospitalization	ICU	Other	Blank	Disease Flare
sJIA							
≤ 1 dose* N = 45	11 (24%)	30 (67%)		1 (2%)**	2 (4%)***	1 (2%)	20 (45%)
2 doses N = 13	3 (23%)	10 (76%)					3 (23%)
3 doses N = 11	1 (9%)	8 (72%)	1 (9%)			1 (9%)	2 (18%)
4 doses N = 1		1 (100%)					
AOSD							
≤ 1 dose* N = 1		1 (100%)					
2 doses N = 3		3 (100%)					1 (33%)
3 doses N = 4		4 (100%)					2 (50%)
4 doses N = 1		2 (100%)					

*Patients who had COVID-19 without any prior doses of the immunization and patients who had COVID after the first dose of a 2-dose immunization regimen.

** This patient later died of disease flare, which was triggered by the infection.

*** Two patients reported that Covid triggered sJIA and MAS.

### Trend towards increased flare with COVID-19 Infection in unimmunized patients

Of the 79 patients who reported a history of COVID-19, we noticed a trend toward a higher risk of flare with infection amongst the un/incompletely immunized patients (20/46 patients − 43%) versus patients who had COVID-19 after at least 2 doses of the immunization (8/33 − 24%). This difference was not statistically significant (p > 0.05). Further confirming this, in a logistic regression including age, sex, age of onset of disease, diagnosis (AOSD vs. sJIA), immunization status, and history of immunization flare or severe side effects with other immunizations, only a history of flare or severe side effects with prior immunization had a statistically significant (p = 0.019) association with the risk of flaring with a COVID-19 infection. Only one of the patients who flared had held their medication for the immunization.

## Discussion

This paper reported on the safety and tolerability of COVID-19 immunization in patients with sJIA and AOSD. The main finding of this study was that the immunization was well tolerated in this population, with very few severe side effects (6 flares, 1 pericarditis, 3 other patients with symptoms lasting ≥ 8 days). The majority of the side effects reported were mild and lasted less than 7 days. This was true even for this population of patients who had a severe phenotype, with a high incidence of MAS (50%) and lung disease (17%), including many patients who had active disease at the time of the immunization (37%).

Our respondents had a similar incidence of systemic side effects than the general population and other autoinflammatory diseases [6–8]. The majority of patients did not hold the medications for the immunization, which is in line with the recommendations from the American College of Rheumatology Guideline for Vaccinations in Patients with Rheumatic and Musculoskeletal Diseases [9]. Only one of the 6 patients who experienced a flare of the disease following the Covid-19 immunization held a medication for the immunization (methotrexate).

There were 6 reports of some type of disease flare with the immunization, out of 99 patients who received the immunization. Of those, all the patients who later contracted COVID-19 also flared with the infection. This suggests that the flare might have been specific to a component of the virus, and not specific to the immunization.

We noticed a trend toward an increased rate of flares of sJIA/AOSD in patients who contracted COVID-19 while unimmunized, which could be a sign that the immunization might have prevented flares with subsequent infection. This finding should be interpreted with caution, since this was not the goal of the study design, and the immunized and unimmunized groups had significant differences at baseline, which could have accounted for the increased rate of flare, including younger age, history of flares with other immunizations, and higher number of medications used in unimmunized patients. In fact, in a logistic regression evaluating risk factors for flaring with a COVID-19 infection, having a history of flare or severe side effects with prior immunization was the only significant association. This suggests that these patients have a disease that is more susceptible to these types of triggers. Nonetheless, further studies should evaluate if COVID-19 immunization prevents disease flare with infection in sJIA.

In our study of sJIA patients, the risk of flare with COVID-19 infection (35% in total in between immunized and non-immunized) was much higher than the risk of flare with the immunization (6%). This is in contrast with the findings of a recent cohort of pediatric rheumatology patients, in which the risk of flare with the vaccine was higher than the risk of flare with the infection (10.7% vs. 3.2% across all diseases) [10]. This retrospective cohort from Thailand included 229 patients with JIA, of which 54 patients had sJIA. In this cohort, they reported that 16.7% of sJIA patients flared after immunization, and only one sJIA patient flared with a COVID-19 infection. It is unclear how many sJIA patients had COVID-19 infection, and if the infections happened before or after immunization.

Another important finding of this study was the evaluation of the reasons for not receiving the immunization. The main reasons for not receiving the COVID-19 immunization were due to concerns with the side effects of the immunization, both specific to sJIA/AOSD and not. There was also significant concern about interaction of the immunization with medications. Of note, some patients reported that the immunization had been contraindicated by a medical provider. Those patients tended to have severe disease, with a history of disease flares with other immunizations.

Although this was not the intention of this survey, we identified 3 patients who reported to have developed AOSD following the COVD-19 immunization, and they were the only patients with hospital admissions for immunization side effects. The diagnosis of AOSD following COVID-19 immunization has been reported in the literature [5], however, given the large number of people who have received the COVID-19 immunization, the risk of development of new onset of AOSD with the immunization is overall low. Interestingly, one of the patients who developed AOSD after his immunization went on to receive boosters of the immunization. The patient did not specify if they developed side effects after the boosters.

One of the strengths of this study was the large number of patients, and the significant representation of patients with more severe spectrum of disease, which are normally under-represented in studies about immunization safety. We also reported the tolerability of the medication on patients who were using a diverse set of medications, including newer medications, such as emapalumab.

The limitations of this study include the fact that this was an anonymous online survey, therefore all the information was self-reported, including the history of sJIA and AOSD. However, given that this survey was posted on closed Facebook groups, in which there is a review of the history of the patients before they are accepted, it is very likely that the population listed in this study indeed corresponded to patients with the correct diagnosis. Studies have also shown that the clinical characteristics of patients recruited through social media are similar to the patients recruited in traditional cohort studies, with some skewing towards patients with rarer conditions [11]. While we did identify a certain skewing towards a more severe phenotype in our cohort compared to other published cohorts of sJIA, with higher rates of MAS and lung disease, this is considered a strength of our study, since this is the group of patients with the most concerns about the safety of the immunization [12]. Our study showed that COVID-19 immunization was tolerated even in patients with a more severe phenotype. Furthermore, some of the information might have been difficult for parents to differentiate, including the differences between immunization side effects, disease flares, and MIS-C. Also, due to limitations with space, the survey did not specifically ask about the timing of the side effects relative to the immunization, which may limit the ability to attribute causality of the side effects. Without detailed clinical and laboratory data, it could also be difficult to determine nuanced information, such as the differentiation of MIS-C / MIS-A from flares of sJIA. The determination of active versus inactive disease was also subjectively determined by the respondents based on their overall impression of the disease status. Another limitation of our study was the lack of clinical and serologic data to verify immunization response, although this was not the goal of this study, which was focused on safety and tolerability. Although the response rate to the survey seemed small compared to the total number of members of the Facebook groups, which could generate a responder bias, there is a large number of non-active members in the group, which overestimates the true number of available respondents.

Furthermore, the survey was also finalized shortly after the COVID-19 immunization was approved for children younger than 5 years of age, so many patients were not eligible for the immunization at the time of the survey, and this population is under-represented in our cohort. It is also notable that the approval of the immunization in the pediatric population, in general, occurred much later than in the adult population. This likely contributed to the significant difference in the age of the immunized and unimmunized groups in this study.

This study shows that COVID-19 immunization was overall well tolerated in patients with sJIA and AOSD, across all spectrums of severity. This study also is a proof of concept that studies can be reliably done with the use of social media for research in patients with sJIA and AOSD, and allow for the capture of patients with severe phenotypes, which are normally under-represented in clinical studies.

## Conclusions

The COVID-19 immunization was well tolerated by sJIA and AOSD patients even in this severe group of patients. There was a low incidence of disease flare with immunization. Most immunization side effects were mild and lasted less than 7 days. The only ICU admission and death from COVID-19 infection occurred in unimmunized subjects.

### Electronic supplementary material

Below is the link to the electronic supplementary material.

Supplementary Material 1

## Data Availability

The datasets used and/or analyzed during the current study are available from the corresponding author upon reasonable request.
