# Supplementary material for: “Online survey of COVID-19 immunization and infection in patients with systemic juvenile idiopathic arthritis and adult-onset still’s disease.”
Source: Pediatr Rheumatol Online J. 2023 Nov 21;21:139. doi: 10.1186/s12969-023-00911-x (PMC10664348; doi:10.1186/s12969-023-00911-x)
Supplement: Supplementary file 1 — Supplementary Material 1 [file 12969_2023_911_MOESM1_ESM.docx]

Supplementary Table 1. Medications used at the time of immunization.

| Medications used at the time of immunization (n=92) | sJIA  N=81 | AOSD  N= 18 |
| --- | --- | --- |
| NSAIDS | 30 (37%) | 3 (17%) |
| Steroids |  |  |
| Oral | 18 (22%) | 6 (33%) |
| IV | 5 (6%) | 0 (0%) |
| IL-1 inhibitors |  |  |
| Anakinra | 18 (22%) | 5 (28%) |
| Canakinumab | 17 (21%) | 1 (6%) |
| Tocilizumab | 16 (20%) | 1 (6%) |
| Methotrexate | 10 (12%) | 1 (6%) |
| JAK inhibitors |  |  |
| Baricitinib | 1 (1%) | 0 (0%) |
| Tofacitinib | 9 (11%) | 0 (0%) |
| Other JAKi | 4 (5%) | 1 (6%) |
| Cyclosporin | 4 (5%) | 0 (0%) |
| Tacrolimus | 5 (6%) | 0 (0%) |
| Abatacept | 4 (5%) | 1 (6%) |
| TNF inhibitors |  |  |
| Adalimumab | 1 (1%) | 1 (6%) |
| Infliximab | 2 (2%) | 0 (0%) |
| IL-18 inhibitor | 1 (1%) | 0 (0%) |
| Hydroxychloroquine | 2 (2%) | 2 (11%) |
| Thalidomide/lenalidomide | 1 (1%) | 0 (0%) |
| Leflunomide | 1 (1%) | 0 (0%) |
| Mycophenolic acid | 0 (0%) | 1 (6%) |
| Paused medication for immunization |  |  |
| Oral steroids (N=18) | 1 (6%) |  |
| Anakinra (N=5) |  | 1 (20%) |
| Methotrexate (N=10) | 6 (60%) |  |
| Tofacitinib (N=9) | 1 (11%) |  |
| Tacrolimus (N=5) | 1 (20%) |  |
| Adalimumab (N=1) |  | 1 (100%) |
| Other JAK inhibitor (N=4) | 1 (25%) |  |
| Leflunomide (N=1) | 1 (100%) |  |

NSAIDs: non-steroidal anti-inflammatory drugs, IL: interleukin, JAK: Janus kinase

Supplementary Table 2. Clinical characteristics of patients with severe* and/or prolonged** side effects from the immunization

|  | Patient 1 | Patient 2 | Patient 3 | Patient 4 | Patient 5 | Patient 6 | Patient 7 | Patient 8 | Patient 9 | Patient 10 |
| --- | --- | --- | --- | --- | --- | --- | --- | --- | --- | --- |
| Age (years) | 55 | 38 | 7 | 8 | 6 | 11 | 67 | 57 | 12 | 11 |
| Sex | Female | Female | Female | Female | Female | Female | Female | Female | Male | Female |
| History of Complications | Arthritis | Arthritis | MAS, sJIA-LD | MAS | MAS, sJIA-LD | Arthritis | MAS, Pericarditis/ myocarditis | Arthritis | Arthritis, Myocarditis/ pericarditis | MAS, Myocarditis/ pericarditis |
| History of flares/severe side effects with other immunizations | Yes | Yes | No | No | No | No | No | No | No | No |
| Medications ever used to treat sJIA | Oral steroids, methotrexate, hydroxychloroquine | NSAIDs, Oral steroids, IV steroids, Anakinra, Tocilizumab, Abatacept, Adalimumab, Etanercept | Oral steroids, IV steroids, Anakinra, Canakinumab, Tocilizumab, Methotrexate, Cyclosporin, Other JAK inhibitor | Oral steroids, IV steroids, Anakinra, Canakinumab, Tocilizumab, Methotrexate, Infliximab | NSAIDs, Oral steroids, IV steroids, Anakinra, Canakinumab, Tofacitinib, Cyclosporin, Other JAK inhibitor | NSAIDs, Oral steroids, Anakinra | NSAIDs, oral steroids | NSAIDs, Oral steroids, Methotrexate | NSAIDs, Oral steroids, IV steroids, canakinumab, Methotrexate | NSAIDs, IV steroids, Anakinra, Cyclosporin, |
| Immunization brand | Moderna and Janssen | Pfizer / BioNTech | Pfizer / BioNTech | Pfizer / BioNTech | Pfizer / BioNTech | Pfizer / BioNTech | Pfizer/ BioNTech and Oxford/ Astra Zeneca | Pfizer/ BioNTech and Oxford/ Astra Zeneca | Pfizer / BioNTech | Pfizer / BioNTech |
| Number of doses | 2 | 3 | 2 | 2 | 2 | 2 | 4 | 4 | 2 | 2 |
| Disease activity at the time of immunization | Inactive disease | Inactive disease | Inactive disease | Inactive disease | Active disease | Inactive disease | Inactive disease | Inactive disease | Inactive disease | Inactive disease |
| Medications used at time of immunization | Hydroxychloroquine | NSAIDs, oral steroids, tocilizumab | Canakinumab | Methotrexate, Infliximab | Oral steroids, IV steroids, Anakinra, Tofacitinib, Cyclosporin | Anakinra | Oral steroids, Mycophenolic Acid | NSAIDs | NSAIDs | None |
| Medications held for immunization | None | None | None | Methotrexate | None | None | None | None | None | N/A |
| Immunization side effects | Fever, Chills, Fatigue/ tiredness  , Myalgia, Rashes, Disease flare | Fever, Chills, Fatigue, Myalgia, Headache, Nausea, Lymphadenopathy Disease flare | None | Flare of Crohn`s disease | Fatigue, tiredness. | Fever, Rashes, Disease Flare | Local symptoms at injection site, Fever, Chills, Fatigue Myalgia, Headache, Nausea, Lymphadenopathy | Local symptoms at injection site, Fatigue, Headache, Rashes, Food sensitivities | Pericarditis | Local symptoms at injection site, Fever, Chills, Fatigue, Myalgia, Headache, Nausea, Lymphadenopathy, Rashes |
| Duration of side effects | 3-7 days | ≥ 8 days | N/A | 3-7 days | 3-7 days | 3-7 days | ≥ 8 days | ≥ 8 days | ≥ 8 days | ≥ 8 days |
| Highest level of care required | Called physician | Called physician | N/A | ED/ urgent care clinic | No medical care was required | Visited physician in clinic | No medical care was required | No medical care was required | ED/ urgent care clinic | Visited physician in clinic |
| Other comments |  |  | Large flare 5 days after second dose |  | Large flare 2 weeks after second dose |  |  |  |  | Diagnosed with new PEG allergy after immunization |

*Severe symptoms defined as any symptoms beyond injection site symptoms, fever, chills, fatigue, myalgias, headache, nausea, lymphadenopathy or rashes). ** Prolonged symptoms defined as lasting 8 days or more.

Supplementary Table 3. New diagnosis of AOSD following immunization

|  | Patient 11 | Patient 12 | Patient 13 |
| --- | --- | --- | --- |
| Age (years) | 35 | 60 | 63 |
| Sex | Female | Female | Male |
| History of Complications | None | MAS | None |
| History of flares/severe side effects with other immunizations | No | Yes | No |
| Medications ever used to treat sJIA | NSAIDS, Oral steroids, IV steroids, Anakinra, Methotrexate, Sulfasalazine | Oral steroids, IV steroids, Anakinra, Tocilizumab, | Oral steroids, IV steroids, Anakinra, Methotrexate, |
| Immunization brand | Oxford / Astra Zeneca | Pfizer / BioNTech and Moderna | Pfizer / BioNTech and Oxford / Astra Zeneca |
| Number of doses | 2 | 3 | 4 |
| Medications used at time of immunization | None | None | Anakinra (only with boosters, did not have disease on first dose) |
| Medications held for immunization | N/A | N/A | None |
| Immunization side effects | Fatigue, Myalgia, lymphadenopathy | Not specified | Fever, MIS-A, Atrial fibrillation |
| Duration of side effects | ≥ 8 days | N/A | ≥ 8 days |
| Highest level of care required | Admitted to the hospital | No medical care was required | ICU admission |
| Other comments | Did not have AOSD until a few days after the immunization | 10 days after Moderna booster patient developed itch in the face, and slowly developed other symptoms: daily fevers, sweats, rash, arthralgia | Patient was given a diagnosis of multi system hyperinflammatory disorder akin to AOSD following administration of Oxford/ Astra Zeneca |

Supplementary table 4. Characteristics of patients with sJIA-associated lung disease by immunization status

|  | **Immunized (n=18)** | **Unimmunized (n=15)** |
| --- | --- | --- |
| Age (years) | 8 (6 -12.5) | 6 (3-10) |
| Age of onset (years) | 1 (1 -2.5) | 1 (1 -2) |
| Female Sex | 16 (88%) | 8 (53%) |
| Country |  |  |
| USA | 17 (94%) | 12 |
| Other | 1 (6%) | 3 |
| Diagnosis |  |  |
| sJIA | 17 (94%) | 15 (100%) |
| AOSD | 1 (6%) |  |
| History of complications |  |  |
| MAS | 15 (83%) | 12 |
| Arthritis | 3 (17 %) | 6 |
| Pericarditis/myocarditis | 1 (6%) | 2 |
| History of disease flare or severe side effects with other immunizations | 0 | 6 |
| NSAIDS | 16 | 12 |
| Steroids | 18 | 15 |
| Oral | 18 | 15 |
| IV | 16 | 13 |
| IL-1 inhibitors |  |  |
| Anakinra | 17 | 15 |
| Canakinumab | 16 | 13 |
| Tocilizumab | 10 | 10 |
| Methotrexate | 11 | 7 |
| JAK inhibitors total |  |  |
| Tofacitinib | 13 | 8 |
| Other JAKi | 7 | 7 |
| Cyclosporin | 12 | 7 |
| Tacrolimus | 5 | 4 |
| Abatacept | 4 | 2 |
| TNF inhibitors |  |  |
| Adalimumab |  | 3 |
| Infliximab | 1 | 2 |
| Emapalumab | 1 | 7 |
| Cyclophosphamide | 2 | 2 |
| Azathioprine | 1 |  |
| Bone marrow transplant | 1 | 1 |
| IL-18 inhibitor | 1 |  |
| IVIG |  | 1 |
| Thalidomide/lenalidomide | 1 |  |
| Mycophenolate Mofetil | 1 |  |
| Outcomes of COVID-19 infection |  |  |
| Never tested positive | 10 | 10 |
| Tested positive at some point | 7 | 4 |
| Asymptomatic | 4 | 1 |
| Mild/moderate symptoms | 3 | 2 |
| Hospitalization in general ward |  |  |
| Hospitalization in ICU |  | 1* |
| Death |  | 1* |
| Flare with infection | 3** | 2 |

sJIA: systemic juvenile idiopathic arthritis, AOSD: adult-onset Still`s disease, NSAIDs: non-steroidal anti-inflammatory drugs, IL: interleukin, JAK: janus kinase. *The patient who was hospitalized in the ICU with the COVID-19 infection passed away 5 months later from a flare triggered by the infection. ** One of the three patients who flared with the infection contracted COVID-19 before the immunization

Supplementary table 5. Patients with severe outcomes of COVID-19 infection

|  | Patient A | Patient B | Patient C | Patient D | Patient E | Patient F | Patient G | Patient H | Patient I | Patient J |
| --- | --- | --- | --- | --- | --- | --- | --- | --- | --- | --- |
| Age (years) | 55 | 20 | 14 | 13 | 2 | 4 | 3 | 68 | 65 | 8 |
| Sex | Female | Female | Female | Male | Male | Female | Female | Female | Female | Male |
| History of Complications | Arthritis | Arthritis | MAS, Arthritis | MAS, sJIA-LD, Arthritis | MAS, sJIA-LD, | Arthritis | MAS, SJIA-LD, arthritis | No | No | MAS |
| History of flares/severe side effects with other immunizations | Yes | No | No | No | Yes | No | No | No | No |  |
| Medications ever used to treat sJIA | Oral steroids, methotrexate, hydroxychloroquine | NSAIDs, Oral steroids, tocilizumab, methotrexate, adalimumab, | NSAIDs, Oral steroids, Anakinra, Canakinumab, Tocilizumab, methotrexate, abatacept, | NSAIDs, Oral steroids, IV steroids, anakinra, canakinumab, methotrexate, tofacitinib, cyclosporin, tacrolimus, cyclophosphamide, azathioprine, IL-18 inhibitor | NSAIDs, oral steroids, IV steroids, anakinra, canakinumab, tocilizumab, ruxolitinib, emapalumab | Oral steroids, methotrexate | NSAIDs, Oral steroids, IV steroids, Anakinra, canakinumab, tocilizumab, tofacitinib, | NSAIDs, oral steroids, IV steroids, adalimumab | NSAIDs, Oral steroids, anakinra, tocilizumab | NSAIDs, Oral steroids, IV steroids, anakinra, tocilizumab, cyclosporin, |
| Immunization brand | Moderna/ Janssen | Moderna | Pfizer/Biontech | Pfizer/Biontech | Not immunized | Not immunized | Not immunized | Pfizer/Biontech | Pfizer/Biontech | Not immunized |
| Immunization status at time of COVID-19 infection | After 2 doses/after 1^st^ Janssen dose | After 2 doses | After 3 doses | After 4 doses | Without immunization | Without immunization | Without immunization | After 3 doses | After 3 doses | Without immunization |
| Symptom severity | Mild/Moderate symptoms | Mild/moderate symptoms | Hospitalized in general ward | Mild/moderate symptoms | Hospitalized in the ICU | Mild/moderate symptoms | Mild/moderate symptoms | Mild/moderate symptoms | Mild/moderate symptoms | Asymptomatic |
| Extra comments | Had two COVID-19 infections. On second infection, had pain, fatigue, vertigo and fevers for 3 weeks | “Developed long term Covid” | “Attacked her liver” | “Coughing is still there after 1 month of covid. Not sure if it has triggered lungs disease” | COVID-19 infection led to several flare ups, ILD, severe MAS, which led to multiple organ failure and death 5 months later | Patient had COVID-19, and had elevated temperatures and fatigue for 4 months, requiring modified school days. Second COVID-19 infection 2 months later, did not cause persistent symptoms | Patient did well with COVID-19 infection. However, developed cough 6 days later, and major flare requiring hospitalization 2 weeks later | Hospitalized for sinus bradycardia possibly caused by COVID-19 infection | Used Paxlovid, with initial response, but had rebound of symptoms after 5 days. Had cough and fatigue for 2 months | Had COVID-19 infection twice. Infection was asymptomatic but had MIS-C 6 weeks after second infection, which triggered a flare that was hard to be controlled. Patient was on unmedicated remission prior to flare and needed to start new meds afterwards. |
